# Reply to “A failure of Fourier transform infrared spectroscopy to type *Burkholderia* isolates from chronically infected patients with cystic fibrosis”

**DOI:** 10.1128/spectrum.02288-23

**Published:** 2023-10-04

**Authors:** K. R. Barker, M. Tadros

**Affiliations:** 1 Division of Microbiology, Department of Laboratory Medicine and Genetics, Trillium Health Partners, Mississauga, Ontario, Canada; 2 Department of Laboratory Medicine and Pathobiology, University of Toronto, Toronto, Ontario, Canada; 3 Institute for Better Health, Trillium Health Partners, Mississauga, Ontario, Canada; 4 Division of Microbiology, Department of Paediatric Laboratory Medicine, Hospital for Sick Children, Toronto, Ontario, Canada; University of Arizona/Banner Health Pathology, Tucson, Arizona, USA

**Keywords:** Fourier-transform infrared spectroscopy, typing, ET12, cystic fibrosis, *Burkholderia*, *Burkholderia cenocepacia*, outbreak, IR biotyper, artificial intelligence, AI

## REPLY

Tkadlec et al*.*, in reference to our study, attempted to verify whether Fourier transform infrared spectroscopy (FTIR) using IR Biotyper would be able to: (i) undertake accurate cluster analysis of *Burkholderia cenocepacia* compared to multilocus sequence typing and (ii) whether repeated isolates of *B. cenocepacia* from the same patient over years, belonged to the same strain (i.e., chronic infection) or were due to different strains (i.e., reinfection). In their brief communication, they concluded that FTIR failed to accurately assign tested isolates to the correct sequence type and thus concluded that the method was not suitable for *B. cenocepacia* strain typing. We have some concerns about the methods of the study that led to this conclusion. The concerns include: (i) the number of replica used per isolate and (ii) the choice of an unsupervised cluster analysis and determined cut-off value.

First, we agree with Tkadlec et al*.* comment regarding the technical difficulty of working with mucoid phenotypes of *Burkholderia* species. We therefore think that running five or six replica of each isolate (as per our study) can improve the accuracy of the IR Biotyper when working with this challenging organism. However, Tkadlec et al*.* noted only three replicates of each isolate.

Second, Tkadlec et al*.* chose unsupervised cluster analysis on IR Biotyper for typing and used a cutoff of 0.2 to determine strain relatedness. We would argue that a supervised cluster analysis is the more robust tool that IR Biotyper offers, with training models, followed by further testing of these models with a different set of strains. These tools such as linear discriminant analysis and artificial neural network utilize artificial intelligence (AI) methods to identify similarities and differences between strains. Another potential limiting factor of the methods was the number of isolates available for typing, *n* = 25. The IR Biotyper AI tools require the creation of a “training model,” followed by “validation” of this model using “unknowns.” Tkadlec et al*.* used a cutoff of 0.2 to determine relatedness. The IR Biotyper instruction manual recommends undertaking multiple exploratory experiments with technical replicates, as well as different organisms to determine a reasonable distance cut-off value to determine which isolates fall into the same cluster and are therefore considered indistinguishable. Some examples include: (i) Hu et al*.* attempted typing of *Klebsiella pneumoniae* on the IR Biotyper (1). Cut-off determination was conducted from three independent experiments on multiple days using whole-genome sequencing as a reference. (ii) Passaris et al*.* attempted serotyping of *Streptococcus pneumoniae* on the IR Biotyper (2). Both supervised and unsupervised cluster analysis (dendograms) were performed. In their study, the cut-off value was chosen manually to separate as many of the different serotypes as possible into different clusters and to group as many strains of the same serotype as possible into one cluster. The chosen cutoff could then be validated using a new set of strains of the species of interest as “unknowns.”

We appreciate the work done by Tkadlec and colleagues, as it adds to the evolving literature on the IR Biotyper. We hope that they might consider acquiring additional well-characterized *B. cenocepacia* isolates and utilization of supervised cluster analysis models for typing this organism.

We think that the IR Biotyper is a very promising tool for rapid and accurate cluster analysis of bacteria. However, we recognize that more work is needed to improve the applications of this tool in hospital epidemiology.
